# Supplementary material for: Selection of suitable wheat genotypes under thermal stress and complex genotype-environment interaction using stability analyses and selection indices
Source: PeerJ. 2025 Sep 25;13:e20061. doi: 10.7717/peerj.20061 (PMC12476864; doi:10.7717/peerj.20061)
Supplement: Supplemental Information 1 [file peerj-13-20061-s001.docx]

**Table S1** Pedigree of the 20 bread wheat genotypes (6 cultivars and 14 doubled haploid lines (DHLs)) used in this study.

| Name | Pedigree |
| --- | --- |
| Gemmeiza-9 | Ald“s”/Huac//CMH74 .630/SxCGM 4583 -5GM- 1GM- OGM |
| Gemmeiza-12 | OTUS/3/SARA/THB//VEEMSS97Y00227S-5y-010M-010Y-010M-2Y-1M-0Y-OGM |
| Sakha-93 | Sakha 92/TR810328 S8871-IS-2S-IS-0S |
| Misr1 | OASSIS / SKAUZ//4*BCN/3/2*PATOR CMSS00Y01881T-050M-030Y-030M-030WGY-33M-0Y-0S |
| Pavone-76 | Vcm//Cno/7C/3/Kal/Bb |
| KSU106 | Barouk/R1474-75-3-53-3-3 |
| DHLs (23,25) | Derived from the cross (Line-115 × Gemmeiza-7) (El-Hennawy et al. 2011) |
| DHLs (5,7,8,11) | Derived from the cross (Line-115 × Giza-164) (El-Hennawy et al. 2011) |
| DHLs (12,14,15,26,29) | Derived from the cross (Gemmeiza-7× Giza-164) (El-Hennawy et al. 2011) |
| DHLs (1,2, 6) | Derived from the cross (Giza-164× Giza-168) (El-Hennawy et al. 2011) |

**Table S2** Monthly agro-climatological data at the experimental location during the growing seasons.

| Parameters  Months | Precipitation (mm) | | | Temperature (°C) | | | | | | | | | Relative Humidity (%) | | |
| --- | --- | --- | --- | --- | --- | --- | --- | --- | --- | --- | --- | --- | --- | --- | --- |
|  |  |  |  | Maximum | | | Minimum | | | Average |  |  |  |  |  |
|  | S1 | S2 | S3 | S1 | S2 | S3 | S1 | S2 | S3 | S1 | S2 | S3 | S1 | S2 | S3 |
| November | 0.31 | 0.27 | 0.17 | 35.37 | 34.82 | 28.07 | 5.05 | 5.84 | 16.37 | 20.21 | 20.33 | 22.03 | 41.69 | 41.21 | 41.34 |
| December | 0.02 | 0.10 | 0.05 | 27.40 | 27.46 | 21.84 | 4.01 | 4.95 | 10.58 | 15.71 | 16.21 | 16.10 | 45.31 | 44.36 | 55.45 |
| January | 0.10 | 0.05 | 0.10 | 29.34 | 28.65 | 19.87 | 1.23 | 2.01 | 8.32 | 15.29 | 15.33 | 14.06 | 40.19 | 40.68 | 47.37 |
| February | 0.00 | 0.00 | 0.00 | 33.79 | 32.43 | 24.66 | 1.21 | 2.08 | 11.62 | 17.50 | 17.26 | 18.19 | 28.44 | 29.26 | 32.89 |
| March | 0.00 | 0.00 | 0.00 | 35.80 | 36.19 | 28.65 | 6.98 | 7.01 | 15.77 | 21.39 | 21.60 | 22.41 | 25.69 | 26.02 | 28.79 |
| April | 0.98 | 1.02 | 0.98 | 39.91 | 38.63 | 33.70 | 14.13 | 15.11 | 21.53 | 27.02 | 26.87 | 27.73 | 31.50 | 30.24 | 31.38 |
| May | 0.01 | 0.00 | 0.01 | 42.91 | 41.77 | 38.97 | 19.21 | 19.94 | 24.87 | 31.06 | 30.86 | 32.37 | 17.69 | 17.05 | 19.69 |

S1, Season 2018/2019; S2, Season 2019/2020, S3, Season 2020/2021.

**Table S3** Grain yield/plot (ton ha^-1^), GY_oc_ and GY_tsc_ under optimal and thermal stress conditions, and stress tolerance indices of different wheat genotypes.

| Variables | GY_oc_ | GY_tsc_ | TOL | SSPI | SSI | YI | RDI | MP | HM | MRP | REI | SWP | ATI | STI | STI_m_ | YSI | GMP | PYR | RSC | SNPI |
| --- | --- | --- | --- | --- | --- | --- | --- | --- | --- | --- | --- | --- | --- | --- | --- | --- | --- | --- | --- | --- |
| G01 | 3.729 | 2.990 | 0.739 | 190.687 | 1.459 | 0.665 | 1.446 | 3.360 | 3.307 | 1.386 | 0.479 | 1.553 | 0.107 | 0.416 | 166.417 | 0.807 | 4.322 | 19.268 | 19.268 | 7.396 |
| G02 | 5.196 | 3.882 | 1.314 | 338.941 | 1.857 | 0.863 | 1.564 | 4.539 | 4.416 | 1.868 | 0.863 | 1.711 | 0.253 | 0.753 | 301.126 | 0.755 | 3.934 | 24.511 | 24.511 | 9.038 |
| G03 | 4.422 | 3.703 | 0.718 | 185.960 | 1.235 | 0.824 | 1.373 | 4.063 | 4.029 | 1.679 | 0.706 | 1.762 | 0.126 | 0.615 | 246.198 | 0.840 | 4.141 | 16.037 | 16.037 | 8.981 |
| G04 | 7.004 | 5.401 | 1.602 | 413.437 | 1.742 | 1.201 | 1.520 | 6.202 | 6.072 | 2.556 | 1.627 | 2.040 | 0.412 | 1.419 | 567.658 | 0.771 | 5.092 | 22.912 | 22.912 | 12.554 |
| G05 | 5.800 | 4.606 | 1.193 | 307.429 | 1.466 | 1.024 | 1.461 | 5.203 | 5.106 | 2.147 | 1.147 | 1.924 | 0.272 | 0.999 | 399.735 | 0.806 | 5.565 | 19.446 | 19.446 | 12.766 |
| G06 | 5.526 | 3.944 | 1.582 | 407.940 | 2.114 | 0.877 | 1.649 | 4.735 | 4.567 | 1.946 | 0.932 | 1.686 | 0.314 | 0.813 | 325.043 | 0.721 | 5.034 | 27.860 | 27.860 | 8.758 |
| G07 | 4.402 | 4.073 | 0.328 | 84.639 | 0.548 | 0.906 | 1.243 | 4.238 | 4.228 | 1.758 | 0.773 | 1.943 | 0.061 | 0.673 | 269.218 | 0.928 | 4.154 | 7.228 | 7.228 | 14.009 |
| G08 | 5.444 | 4.948 | 0.496 | 128.089 | 0.661 | 1.101 | 1.266 | 5.196 | 5.177 | 2.154 | 1.159 | 2.124 | 0.114 | 1.010 | 404.154 | 0.913 | 4.705 | 8.712 | 8.712 | 16.867 |
| G09 | 5.955 | 4.817 | 1.139 | 293.536 | 1.388 | 1.071 | 1.433 | 5.386 | 5.303 | 2.223 | 1.231 | 1.981 | 0.265 | 1.073 | 429.313 | 0.816 | 5.424 | 18.358 | 18.358 | 12.511 |
| G10 | 5.379 | 5.125 | 0.253 | 65.839 | 0.355 | 1.140 | 1.207 | 5.252 | 5.246 | 2.181 | 1.186 | 2.211 | 0.057 | 1.034 | 413.689 | 0.955 | 5.089 | 4.531 | 4.531 | 49.070 |
| G11 | 4.177 | 3.562 | 0.615 | 159.208 | 1.123 | 0.792 | 1.348 | 3.869 | 3.844 | 1.600 | 0.641 | 1.743 | 0.103 | 0.559 | 223.583 | 0.854 | 4.625 | 14.608 | 14.608 | 8.807 |
| G12 | 5.293 | 3.894 | 1.399 | 360.870 | 1.965 | 0.866 | 1.589 | 4.593 | 4.462 | 1.890 | 0.884 | 1.698 | 0.271 | 0.771 | 308.205 | 0.741 | 4.337 | 25.933 | 25.933 | 8.646 |
| G13 | 5.144 | 3.339 | 1.805 | 465.644 | 2.637 | 0.742 | 1.837 | 4.241 | 4.010 | 1.737 | 0.736 | 1.476 | 0.314 | 0.642 | 256.807 | 0.653 | 4.464 | 34.668 | 34.668 | 6.854 |
| G14 | 4.373 | 3.062 | 1.311 | 338.647 | 2.307 | 0.681 | 1.651 | 3.717 | 3.600 | 1.526 | 0.582 | 1.461 | 0.207 | 0.508 | 203.071 | 0.698 | 3.817 | 30.164 | 30.164 | 6.174 |
| G15 | 6.560 | 3.589 | 2.972 | 767.100 | 3.457 | 0.797 | 2.284 | 5.074 | 4.560 | 2.065 | 1.014 | 1.399 | 0.589 | 0.886 | 354.426 | 0.546 | 4.473 | 45.419 | 45.419 | 6.919 |
| G16 | 4.821 | 3.525 | 1.296 | 334.952 | 2.059 | 0.784 | 1.585 | 4.173 | 4.066 | 1.716 | 0.733 | 1.603 | 0.228 | 0.640 | 256.021 | 0.730 | 4.126 | 26.989 | 26.989 | 7.380 |
| G17 | 3.837 | 2.770 | 1.067 | 275.168 | 2.089 | 0.616 | 1.617 | 3.304 | 3.203 | 1.358 | 0.457 | 1.417 | 0.148 | 0.398 | 159.351 | 0.725 | 3.669 | 27.495 | 27.495 | 5.970 |
| G18 | 6.723 | 5.901 | 0.822 | 211.888 | 0.903 | 1.313 | 1.315 | 6.312 | 6.275 | 2.613 | 1.705 | 2.279 | 0.224 | 1.487 | 594.602 | 0.881 | 4.302 | 11.927 | 11.927 | 17.998 |
| G19 | 6.990 | 5.093 | 1.897 | 489.369 | 2.055 | 1.132 | 1.619 | 6.042 | 5.857 | 2.484 | 1.528 | 1.927 | 0.472 | 1.334 | 533.484 | 0.729 | 6.421 | 27.064 | 27.064 | 11.226 |
| G20 | 5.012 | 4.768 | 0.244 | 62.967 | 0.367 | 1.061 | 1.209 | 4.890 | 4.886 | 2.031 | 1.030 | 2.130 | 0.052 | 0.898 | 359.033 | 0.952 | 5.030 | 4.775 | 4.775 | 21.468 |
